# Optimization of iron electrocoagulation parameters for enhanced turbidity and chemical oxygen demand removal from laundry greywater

**DOI:** 10.1038/s41598-024-67425-8

**Published:** 2024-07-16

**Authors:** Ibrahim Tabash, Haitham Elnakar, Muhammad Faizan Khan

**Affiliations:** 1 Department of Civil and Environmental Engineering, King Fahd University of Petroleum & Minerals, 31261 Dhahran, Saudi Arabia; 2 Interdisciplinary Research Center for Construction and Building Materials, King Fahd University of Petroleum & Minerals, 31261 Dhahran, Saudi Arabia; 3 Department of Bioengineering, King Fahd University of Petroleum & Minerals, 31261 Dhahran, Saudi Arabia

**Keywords:** Greywater treatment, Iron electrocoagulation, Response surface methodology (RSM), Central composite design (CCD), Turbidity removal, COD removal, Chemical engineering, Civil engineering

## Abstract

This study explores the optimization of iron electrocoagulation for treating laundry greywater, which accounts for up to 38% of domestic greywater. Characterized by high concentrations of surfactants, detergents, and suspended solids, laundry greywater presents complex challenges for treatment processes, posing significant environmental and health risks. Utilizing response surface methodology (RSM), this research developed a second-order polynomial regression model focused on key operational parameters such as the area-to-volume ratio (A/V), current density, electrolysis time, and settling time. Optimal treatment conditions were identified: an A/V ratio of 30 m^2^/m^3^, a current density of 10 mA/cm^2^, an electrolysis duration of 50 min, and a settlement period of 12 h. Under these conditions, exceptional treatment outcomes were achieved, with turbidity removal reaching 94.26% and COD removal at 99.64%. The model exhibited high effectiveness for turbidity removal, with an R^2^ value of 94.16%, and moderate effectiveness for COD removal, with an R^2^ value of 75.90%. The interaction between the A/V ratio and electrolysis time particularly underscored their critical role in electrocoagulation system design. Moreover, these results highlight the potential for optimizing electrocoagulation parameters to adapt to daily fluctuations in greywater production and meet specific household reuse needs, such as toilet flushing. This tailored approach aims to maximize contaminant separation and coagulant efficiency, balance energy use and operational costs, and contribute to sustainable water management.

## Introduction

Amidst the escalating global challenge of water scarcity, which affects over two billion individuals deprived of access to safe drinking water^1^, the valorization of greywater emerges as a pivotal strategy. Greywater, emanating from domestic activities such as laundry, bathing, and dishwashing, holds the potential for reuse in non-potable applications, including irrigation and toilet flushing^2^, thereby alleviating the demand on freshwater resources and diminishing the load on conventional water treatment infrastructures. Particularly, Laundry greywater represents a substantial portion, up to 38%, of a household's total greywater, and is characterized by high levels of surfactants, detergents, and suspended solids which complicate treatment processes and pose environmental and health risks if not properly managed^3–5^. The variable daily generation of greywater highlights the need for adaptable and efficient treatment systems. For example, in some cases, only laundry water may be required for specific reuse applications such as toilet flushing, reducing the volume that needs comprehensive treatment. This variability underscores the importance of optimizing treatment processes to ensure a consistent supply of high-quality treated water, tailored to the fluctuating demands of a household or community.

Greywater treatment technologies encompass a range of methods designed to purify water from household sources, excluding toilet waste, making it suitable for reuse. Common approaches include biological treatments, filtration, sedimentation, and chemical coagulation, each with its specific utility and limitations based on the greywater's composition and the desired quality of the treated water^6–8^. Electrocoagulation (EC) stands out among these methods due to its versatility and efficiency^9,10^. Unlike traditional coagulation that relies on chemical dosing, EC induces coagulation electrochemically, offering precise control over the process without the addition of external chemicals, thus reducing the risk of secondary pollution^11^. This process effectively destabilizes and aggregates contaminants, facilitating their removal^12^. Moreover, EC's adaptability to varying water qualities and its ability to tackle a broad spectrum of pollutants, from organic compounds to heavy metals, gives it a significant advantage, particularly in settings where water quality can fluctuate^13^ These attributes make electrocoagulation a compelling choice for greywater treatment, aligning with sustainability goals by minimizing chemical usage and enhancing treatment efficacy.

Furthermore, the optimization of iron electrocoagulation parameters like electrode area-to-volume ratio (A/V), current density, electrolysis time, and settling time plays a critical role in improving the process's efficiency and sustainability^14–16^. Optimizing these parameters not only enhances the availability of coagulants and the separation of contaminants, leading to improved water clarity and quality, but it also helps in managing energy consumption, striking a balance between effectiveness and operational costs. Socio-economic characteristics also influence greywater generation rates and volumes, as households with varying sizes and economic statuses exhibit different water usage patterns and capacities for water reuse, making the customization of greywater treatment systems based on these characteristics essential for maximizing efficiency and sustainability^17–20^.

Focusing on laundry greywater, especially that colored with blue dye, this research aims to dissect the electrocoagulation process's efficacy, employing iron and stainless-steel electrodes. The study is dedicated to unraveling the effects of crucial operational parameters—A/V ratio, current density, settling time, and electrolysis duration—on turbidity and COD removal. Leveraging Response Surface Methodology (RSM) for experimental design and analysis, the research endeavors to pinpoint optimal conditions for maximizing contaminant elimination. This inquiry not only contributes to advancing sustainable wastewater treatment technologies but also offers insights for refining and enhancing greywater recycling solutions, fostering the development of environmentally harmonious wastewater management practices.

## Materials and methodology

### Greywater treatment experimental setup

Blue-like greywater samples were collected from the washing of blue clothes after the completion of the washing cycle of an automatic washing machine from a residential building located in Dhahran, Saudi Arabia. The physicochemical characteristics of the samples are shown in Table 1. Experiments were conducted shortly after sample collection to capture the immediate post-washing state, thereby minimizing potential alterations in composition that could arise from extended storage or exposure to external conditions.

**Table 1:** Characteristics of laundry greywater samples.

Parameter	Unit	Mean
pH	–	6.17
Turbidity	NTU	218
Conductivity	µS/cm	572
Alkalinity	mg/L as CaCO_3_	71
TOC	mg/L	345
TSS	mg/L	445
TDS	mg/L	269
COD	mg/L	1535
Aluminum	mg/L	0.2
Arsenic	mg/L	0.55
Cobalt	µg/L	19
Cadmium	µg/L	6
Copper	µg/L	6
Calcium	mg/L	7
Chromium	µg/L	10
Boron	mg/L	0.9
Barium	µg/L	2
Mercury	µg/L	15
Potassium	mg/L	14
Magnesium	mg/L	0.25
Manganese	µg/L	10
Sodium	mg/L	10
Nickel	µg/L	1
Lead	mg/L	0.25
Selenium	µg/L	3
Silicon	mg/L	0.2
Zinc	mg/L	0.1

A 1000-mL glass beaker was used as the electrochemical reactor. The selection of sample volume and submerged surface area for experimentation was based on an A/V ratio falling within the range of 15–45 m^2^/m^3^. Within the beaker, two iron electrodes as anode and two stainless-steel electrodes as cathode were placed, each measuring 11 cm in length, 5 cm in width, and 0.2 cm in thickness. These electrodes were positioned parallel to each other, maintaining a consistent separation distance of 3 cm. In order to prevent the possibility of further dissolution from the cathode, a stainless-steel cathode was used. The experiments were conducted under standard ambient conditions of temperature (25 °C). A magnetic stirrer was positioned at the midpoint of the reactor. Prior to applying electric current, samples were stirred for 5 min to ensure uniformity. The stirrer operated at a speed of approximately 500 rpm, ensuring vigorous mixing within the EC reactor^21–25^. The DC-regulated power source supplied the electric current. The electric current was supplied by a DC-regulated power source (Wanptek, DPS 3010U, 1–30 V DC and 10 A, China). Different current densities were tested, each applied for varying durations. Each treatment duration constituted an independent experiment. At the conclusion of each treatment time, both the DC-regulated power source and stirring were halted, followed by a different settling time. Subsequently, samples were collected from the clear layer beneath the surface froth layer.

### Analytical methods

Turbidity levels were measured utilizing a turbidimeter (Model 2100AN, HACH, USA). The pH values of the greywater samples were gauged with a calibrated pH meter (Model pH700, OAKTON, USA). Total Organic Carbon (TOC) concentrations were measured employing a high-temperature combustion analyzer (Model TOC-VCSN, SHIMADZU, Japan). Measurements of chemical oxygen demand (COD), alkalinity, total suspended solids (TSS), and total dissolved solids (TDS) were carried out in accordance with Standard Methods^26^. The electrical conductivity, indicative of the ion concentration within the solution, was determined using a conductivity meter (Model Accumet XL 30, Fisher Scientific, USA). The analysis of UV absorbance at 254 nm was executed with a UV spectrophotometer (Model UV-1601PC, SHIMADZU, Japan). The presence of metals in the greywater samples was evaluated through Inductively Coupled Plasma-Mass Spectrometry (ICP-MS) (Model iCAP6300 Duo, Thermo Scientific, USA).

### Experimental design

The application of Response Surface Methodology (RSM) was undertaken through the utilization of Central Composite Design (CCD). The implementation of CCD is instrumental in achieving rotatability for both linear and quadratic terms, offering significant benefits for the optimization and exploration of the interrelations among multiple independent variables and one or more dependent variables. The experimental design was structured to investigate the influences of four principal factors: the A/V ratio (X_1_), current density (X_2_), electrolysis time (X_3_), and settling time (X_4_). The experimental conditions for these four variables (X_1_, X_2_, X_3_, and X_4_) are encapsulated in Table 2, with each variable being evaluated at three distinct levels, denoted as − 1, 0, and 1, corresponding to low, medium, and high values, respectively.

**Table 2:** Levels of the factors tested in the central composite design.

Independent factors	Units	Symbol	Coded and absolute levels		
			− 1	0	1
A/V ratio	m^2^/m^3^	X_1_	15	30	45
Current density	mA/cm^2^	X_2_	5	10	15
Electrolysis time	mins	X_3_	10	30	50
Settling time	hrs	X_4_	1	12	23

The influence of the independent variables on two predetermined response variables was scrutinized through the implementation of 28 experiments, as formulated by RSM. These response variables were turbidity and COD. The experimental design matrix is delineated in Table 3. A second-order nonlinear regression model, encapsulated by a quadratic polynomial equation, was employed to fit the response variables, as depicted in Eq. (1):

1 $$Y= {\upbeta }_{0}+\sum_{i=1}^{k}{\upbeta }_{i}{x}_{i}+\sum_{i=1}^{k}{\upbeta }_{ii}{x}_{i}^{2}+\sum_{i<j}^{k}{\upbeta }_{ij}{x}_{i}{x}_{j}+\varepsilon$$

where: Y is the response variable, X_i_​ and X_j_ are the coded levels of the factors, β_0_, β_i_, β_ii_, β_ij_ are the model coefficients, k is the number of factors, ε is the error term.

**Table 3:** The Central Composite Design experimental design matrix of four variables along with the related experimental and calculated response.

Run	X_1_: A/V ratio (m^2^/m^3^)	X_2_: Current density (mA/cm^2^)	X_3_: Electrolysis time (mins)	X_4_: Settlement time (hrs)	Measured turbidity removal (%)	Modeled turbidity removal (%)	Measured COD removal (%)	Modeled COD removal (%)
1	45	5	10	1	87.03	87.46	96.52	99.77
2	30	5	30	12	90.28	87.31	98.65	99.46
3	45	15	10	23	93.79	94.73	99.65	100
4	45	15	10	1	90.72	87.68	99.00	93.46
5	45	5	10	23	89.92	91.06	98.38	98.42
6	30	15	30	12	90.75	87.99	98.27	96.17
7	15	15	10	1	21.84	26.08	61.89	71.87
8	30	10	30	12	85.10	89.58	97.31	98.51
9	15	5	10	1	35.81	38.94	93.70	87.57
10	30	10	30	1	82.29	77.97	96.66	95.16
11	15	5	50	1	77.31	80.23	97.02	100
12	30	10	30	23	87.18	85.77	97.65	97.87
13	45	15	50	23	81.70	76.50	92.12	94.50
14	30	10	30	12	85.10	89.58	97.31	98.51
15	45	15	50	1	75.29	79.84	93.40	94.27
16	45	10	30	12	83.79	90.14	93.54	97.24
17	15	5	10	23	58.56	57.87	89.48	92.76
18	30	10	50	12	94.26	95.91	99.65	100
19	15	10	30	12	84.67	72.59	97.92	92.90
20	30	10	30	12	85.10	89.58	97.99	98.51
21	45	5	50	1	73.02	69.06	93.86	93.74
22	15	15	10	23	46.55	48.46	89.28	85.66
23	30	10	30	12	85.10	89.58	97.31	98.51
24	15	15	50	23	86.48	89.92	97.11	98.03
25	30	10	10	12	91.76	84.38	99.64	97.42
26	45	5	50	23	62.64	62.27	91.18	85.37
27	15	5	50	23	87.78	88.76	96.50	98.29
28	15	15	50	1	81.13	77.93	95.03	91.25

The experimental parameters and responses were subjected to evaluation via Design Expert software, version 12 (Stat-Ease, USA). To assess the adequacy of the constructed model, Analysis of Variance (ANOVA) was utilized for diagnostic testing. The statistical relevance of the model under consideration, as well as the parameters incorporated within, was scrutinized through the application of the F-value and p-value, with a confidence interval set at 95%. Moreover, the congruence of the multi-regression model was quantified by the coefficient of determination (R^2^) and the standard error associated with the estimate.

## Results and discussion

### Laundry greywater characteristics

Laundry greywater, as indicated by Table 1, has distinct physicochemical parameters. The mean pH level for laundry greywater presented in Table 1 was 6.17, which is slightly acidic. A similar pH of 6.79 was also observed in laundry greywater collected from student housing at Kenyatta University, Kenya^27^. Iron electrocoagulation is a suitable technology to treat this laundry greywater as the optimal pH range for iron electrocoagulation is typically between 6.0 and 8.0. Within this range, iron electrodes are most effective at generating iron hydroxide flocs, which facilitates the removal of contaminants^28^. The turbidity of the laundry greywater, measured at 218 NTU, falls within the range of 14 to 400 NTU for domestic laundry greywater as reported by Ref.^29^. Greywater specifically from laundry sources is expected to have higher turbidities due to the presence of TSS from clothes in addition to the source water used for washing. Fabric fibers and zeolites from detergents also contribute to higher TSS concentrations in laundry greywater^30,31^. As noted in Table 1, a TSS concentration of 445 mg/L was observed in the turbid laundry greywater collected in this study.

Laundry greywater usually contains different substances originating from organic and inorganic sources. Organics can include grease, soaps, detergents, biological substances, and fat, whereas metal ions, heavy metals, sand, and dust may constitute inorganic or emerging substances^29,32,33^. Therefore, laundry greywater from very dirty items can have COD values as high as 20,000 mg/L while greywater generated from typical households can have COD concentrations in the range of 400–1200 mg/L^34^. A higher-than-average COD concentration of 1,535 mg/L observed in this study could be due to the blue dye from the blue-colored clothes utilized for washing. Similarly, the TOC concentration of 345 mg/L observed in this study appears to be higher than some of the previously reported studies, including studies reporting as low as 86 mg/L^35^. This suggests that the laundry greywater collected in this study constitutes a significant organic load, which, if untreated, could contribute to biological growth and oxygen depletion in receiving environments.

Table 1 also presents a range of metal concentrations, from common elements like sodium and potassium to heavy metals such as arsenic and mercury, albeit at low concentrations. It is noted that the presence of boron can be problematic when reuse for crop irrigation is considered for laundry greywater. If present, boron ions can be taken up by plants and accumulate to concentrations high enough to cause crop damage or reduce overall yield^36^. The observed concentration of boron in this study was 0.9 mg/L, which is slightly below the recommended value of 1.0 mg/L for irrigating sensitive crops such as lemon, onion, and beans^37^.

Compared to other sources of greywater, such as those from bathing or kitchens, laundry greywater typically contains higher concentrations of detergents and surfactants, and, as emphasized in this study, potentially problematic dyes^38^. These contaminants can be more challenging to treat due to their synthetic nature and propensity to interact with other substances. Unlike kitchen greywater, which may have higher levels of organic material and nutrients, laundry greywater's challenge lies in the chemical complexity rather than the biological content. Laundry detergents typically contain a variety of chemicals that are designed to remove stains, dirt, and odors from clothing. The presence of these chemicals adds complexity to the greywater composition and can affect its properties such as pH, foamability, and biodegradability^30^. In comparison to broader wastewater matrices, which include sewage and industrial effluents, laundry greywater is typically less contaminated with pathogens, mainly arising from washing fecal-contaminated clothes^39^. However, it may contain similar levels of certain pollutants, such as surfactants and heavy metals, depending on the source. The concentration of these pollutants in laundry greywater can be significant, particularly when considering the cumulative effect of continuous discharge. Therefore, laundry greywater requires careful consideration and appropriate treatment before reuse to ensure public health protection and environmental sustainability.

### Treatability of laundry greywater

The present study demonstrated an optimum turbidity removal efficiency of 94.26%, with values ranging from 21.84% to 94.26%, and a COD removal efficiency of 99.65%, with an initial concentration of 1758 mg/L (Table 3). Maximum treatment efficacy was achieved under optimal conditions: an A/V ratio of 30 m^2^/m^3^, a current density of 10 mA/cm^2^, an electrolysis duration of 50 min, and a settlement period of 12 h. These optimized conditions resulted in exceptional treatment outcomes, with turbidity removal reaching 94.26% and COD removal at 99.65%. The robustness of these results was confirmed through confirmatory experiments conducted in triplicate, consistently yielding similar outcomes with a variation of ± 1% for turbidity and ± 0.5% for COD. The effectiveness of both turbidity and COD removal varied significantly across the studies analyzed, as shown in Table 4^40–50^. The study by Bani-Melhem and Rasool Al-Kilani (2023) achieved similar results for turbidity removal using iron and mild steel electrodes, reporting efficiencies between 92 and 94%^40^. However, their initial turbidity concentration was significantly higher (506 NTU), and their COD removal rates were 86.5% and 85.3% for different electrode materials, starting from an initial concentration of 1290 mg/L.

**Table 4:** Comparative analysis of greywater treatment using electrocoagulation in different studies.

Study parameters		Current Study	Study 1^40^	Study 2^41^	Study 3^42^	Study 4^43^	Study 5^44^	Study 6^45^	Study 7^46^	Study 8^47^	Study 9^48^	Study 10^49^	Study 11^50^
Electrode material	Anode	Iron	Iron and mild steel	Aluminum	Aluminum	Aluminum and Iron	Aluminum	Aluminum and Iron	Iron and Aluminum	Stainless Steel	Aluminum	Aluminum	Aluminum
	Cathode	Stainless steel		Iron									
pH	Optimum value	6.26	7.8	4.67	7	7.5	5.1	7.4	7	5.4	7.65	6.5	8.3
	Range	6.18–7.21	N/A	2–12	3–11	4–10	3.64–10.36	6.3–8.1	3–11	2–11	6.2–9.4	6.5–7.1	3–10
A/V ratio (m^2^/m^3^)	Optimum value	30	N/A	N/A	N/A	N/A	N/A	N/A	N/A	N/A	N/A	﻿N/A	N/A
	Range	15–45											
Current density (mA/cm^2^)	Optimum value	10	5 (Fe) and 10 (M)	16.25	0.3	0.5	3.95	3	15	4.68	0.2	12.5	N/A
	Range	5–15	5–20	4.7–23.4	0.1–0.5	0.5–30	1.3–4.62	0.6–3	5–20	0.52–10.58	10–50	3.1–18.8	0–1.6
Electrolysis time (minutes)	Optimum value	50	10	31.67	60	30	53.5	40	60	55	60	30	60
	Range	10–50	N/A	10–50	10–90	N/A	9.55–110.45	5–40	10–60	15–60	N/A	10–90	15–90
Settling time (hours)	Optimum value	12	0.033	N/A	N/A	N/A	60 min	N/A	N/A	N/A	N/A	0.33	1
	Range	1–23	N/A				N/A					N/A	N/A
Turbidity removal (%)	Initial concentration (NTU)	231	506	150–500	N/A	15.1	118–126	60.2 ± 19	242 ± 5	43.9	104	37.83	245
	Optimum value (%)	94.26	92 (M), 94 (Fe)	90.9		98 (Al), 78 (Fe)	90.5	97 (Al), 99 (Fe)	91.65 (Fe)	N/A	85	92.6	95.9
	Range (%)	21.84–94.26	92–100 (M), 84–98 (Fe)	76.12–94.78		98–100 (Al), 78–88 (Fe)	55–98.75	65–97 (Al), 97–99 (Fe)	N/A	N/A	N/A	13.6–92.6	88–95.9
COD removal	Initial concentration (mg/L)	1758	1290	1300–2000	690 ± 25	470	670–677	207 ± 32	460 ± 50	334.13 ± 3.8	520	163 ± 82	4155
	Optimum value (%)	99.65	86.5 (M), 85.3 (Fe)	96.1	70	87 (Al), 84 (Fe)	84.4	88 (Al) and 79 (Fe)	85 (Fe), 72 (Al)	98.56 ± 0.14	70	52.8	93.2
	Range (%)	61.89–99.65	86–87 (M), 77–85.3 (Fe)	74.36–99.99	28–71.2	87–89 (Al), 84–87 (Fe)	49.94–94.99	70–88 (Al), 55–79 (Fe)	30–85 (Fe), 22–72 (Al)	68.03–98.47	N/A	N/A	80–93.2

Mousazadeh et al. reported an optimum turbidity removal of 90.9% using aluminum and iron electrodes, across a wide range of initial concentrations (150–500 NTU) and achieved a COD removal efficiency of 96.1% with initial concentrations ranging from 1300 to 2000 mg/L^41^. Additionally, the studies by Shakeri et al.^44^ and Çalışkan et al.^45^ underscored the high efficacy of aluminum and iron electrodes, achieving optimum turbidity removals of 97–99% and 55–98.75%, respectively, and COD removal efficiencies ranging from 49.94 to 94.99%. Interestingly, Jung et al. reported one of the highest COD removal efficiencies at 98.56% using stainless-steel electrodes, indicating that stainless-steel, when optimized, can be highly effective in COD removal, despite its comparatively lower performance in turbidity removal^47^. Conversely, studies such as Nasr et al. (2016) reported a lower COD removal efficiency of 52.8%^50^. These findings suggest that while various electrode materials and configurations can achieve high removal rates for both turbidity and COD, the specific choice and optimization of operational parameters are crucial for maximizing efficiency. Iron, aluminum, and stainless-steel electrodes have all demonstrated significant removal capabilities; however, achieving near-complete removal necessitates careful tuning of the process conditions. The high variability in results underscores the need for further research to standardize and optimize electrocoagulation processes for different types of wastewater.

### Experimental design analysis

#### Turbidity response factor

Table 5 shows the ANOVA results for turbidity and COD removal. The R^2^ value for turbidity removal is remarkably high at 94.16%, indicating an excellent fit of the regression model to the observed data. This value suggests that 94.16% of the variability in turbidity removal is accounted for by the independent variables in the model. Such a high R^2^ value signifies that the model has robust predictive power and that the factors chosen for the study are highly indicative of the outcomes for turbidity reduction in the treatment process. The sum of squares for the model stands at 8041.56, which is a direct measure of the total variation that the model explains. In contrast, the error sum of squares, at 499.16, is considerably lower, indicating that the unexplained variance, or noise, is minimal relative to the explained variance. The mean square for the model is 574.40, and the error mean square is 38.40. The large difference between these two values suggests a significant effect of the model over the random error, further confirming the model's efficacy. The F-statistic, at 14.96, derived from the ratio of the model mean square to the error mean square, along with the p-value being less than 0.05, indicates that the model is extremely significant. The p-value essentially rejects the null hypothesis, which posits that the model has no explanatory power, affirming the validity of the model in elucidating the treatment process for turbidity.

**Table 5:** Analysis of variance (ANOVA) results for Turbidity and COD removal efficiency.

Response		Sum of square	Mean square	*F*- statistic	*p*-value
Turbidity R^2^ = 94.16	Model	8041.56	574.40	14.96	< 0.0001
	Error	499.16	38.40	3.99	–
	Total	8540.72	–	–	–
COD R^2^ = 75.90	Model	1033.84	73.85	2.92	0.0305
	Error	328.26	25.25	–	–
	Total	1362.10	–	–	–

**Figure 1:**
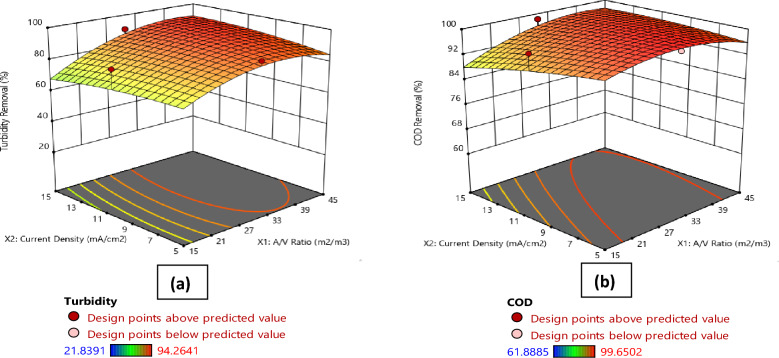
Comparative 3D surface plots depicting the influence of current density and area to volume ratio (A/V) on (**a**) Turbidity Removal and (**b**) COD Removal percentage.

**Figure 2:**
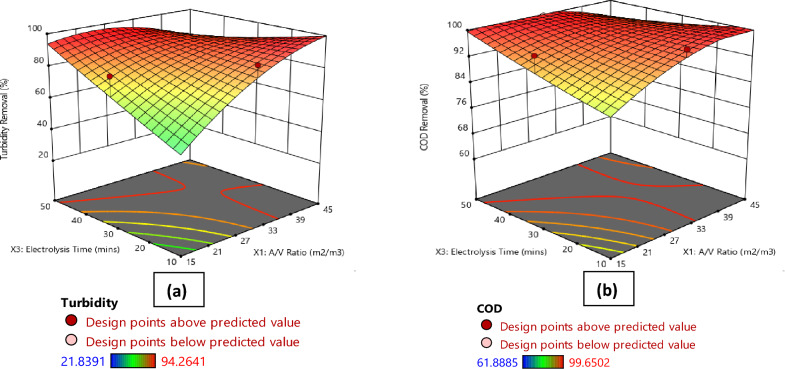
Comparative 3D surface plots depicting the influence of electrolysis time and area to volume ratio (A/V) on (**a**) Turbidity Removal and (**b**) COD Removal percentage.

**Figure 3:**
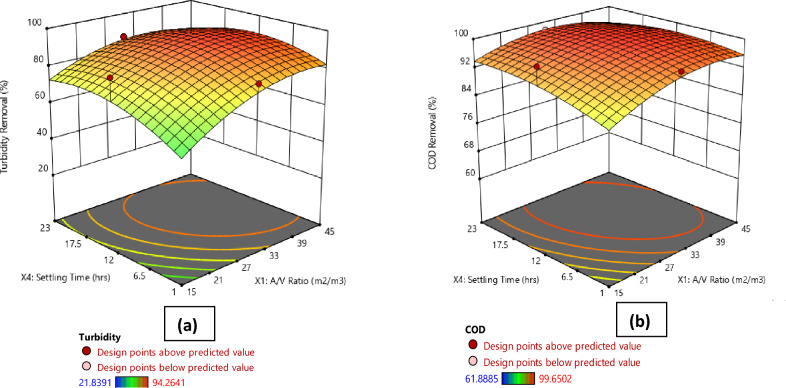
Comparative 3D surface plots depicting the influence of settling time and area to volume ratio (A/V) on (**a**) Turbidity Removal and (**b**) COD Removal percentage.

**Figure 4:**
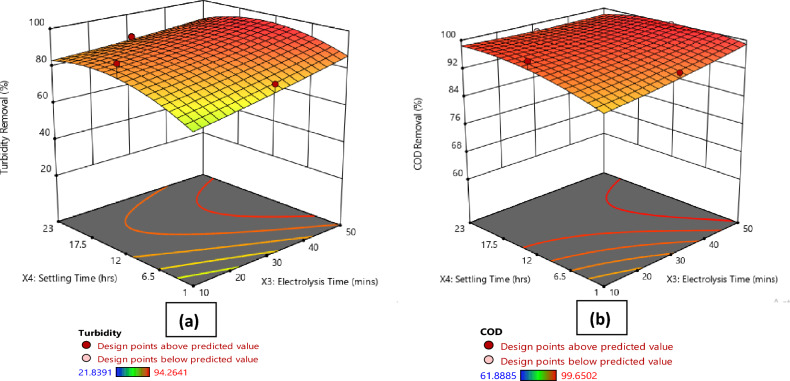
Comparative 3D surface plots depicting the influence of settling time and electrolysis time on (**a**) Turbidity Removal and (**b**) COD Removal percentage.

Figures 1, 2, 3, 4a present the 3D surface plots depicting the influence of the interactions between the principal parameters selected in this study on turbidity removal efficiency. In Fig. 1a, the 3D surface plot illustrates the influence of current density and A/V ratio on turbidity removal percentage. It is observed that the turbidity removal efficiency increases with higher current densities. Furthermore, the A/V ratio also plays a significant role, with optimal values enhancing the turbidity removal process. Moving to Fig. 2a, the effect of electrolysis time and A/V ratio on turbidity removal is depicted. The results indicate that increasing the electrolysis time generally improves turbidity removal efficiency, and the A/V ratio further enhances this effect, with the highest removal rates observed at longer electrolysis times and optimal A/V ratios. Similarly, Fig. 3a shows the influence of settling time and A/V ratio on turbidity removal, where longer settling times lead to higher turbidity removal percentages, highlighting the importance of sufficient sedimentation time in the process. The A/V ratio also contributes positively, with the highest removal efficiencies occurring at optimal values. Finally, Fig. 4a demonstrates the combined effects of settling time and electrolysis time on turbidity removal. Both parameters positively influence the removal efficiency, with extended times for both electrolysis and settling resulting in the highest turbidity removal rates, indicating that an adequate balance of both time factors is crucial for optimal turbidity removal.

The response function for the turbidity removal efficiency (%) is shown in Eq. (2) in terms of the coded levels of the factors. Equation (2) includes only those terms that were found to be significant, very significant, or marginally significant based on their p-values and F-values (Table 6). The terms X_1_ (A/V Ratio), X_3_ (Electrolysis Time), and X_4_ (Settlement Time) were included due to their strong statistical significance, with p-values of less than 0.05 and F-values of 36.02, 15.54, and 7.12, respectively. The interaction terms X_1_X_3_ and X_1_X_4_ were also included, as they showed very significant effects (F-value = 92.80, p-value < 0.05) and significant effects (F-value = 6.12, p-value = 0.0280), respectively. Additionally, the quadratic term X_1_^2^ and interaction term X_1_X_2_ were included due to their marginal significance (F-values of 4.53 and 4.46, p-values of 0.0529 and 0.0547, respectively), reflecting their potential impact on the model. Lastly, the quadratic term X_4_^2^ was included due to its marginal significance (F-value = 3.99, p-value = 0.0670).

2 $${\text{Turbidity}} {\text{Removal}} {\text{Efficiency}} \left( \% \right) = {89}.{51} + {8}.{\text{77X}}_{{1}} + {5}.{\text{76X}}_{{3}} + {3}.{9}0{\text{X}}_{{4}} - {14}.{\text{92X}}_{{1}} {\text{X}}_{{3}} - {3}.{\text{83X}}_{{1}} {\text{X}}_{{4}} - {8}.{\text{22X}}_{{1}}^{{2}} + {3}.{\text{27X}}_{{1}} {\text{X}}_{{2}} - {7}.{\text{71X}}_{{4}}^{{2}}$$

**Table 6:** ANOVA results for turbidity and COD removal.

Source	Turbidity removal				COD removal			
	Sum of squares	Mean square	F-value	*p*-value	Sum of squares	Mean square	F-value	*p*-value
Model	8041.56	574.40	14.96	< 0.05	1033.84	73.85	2.92	< 0.05
X_1_-A/V ratio	1382.93	1382.93	36.02	< 0.05	87.71	87.71	3.47	0.0851
X_2_-current density	1.93	1.93	0.0503	0.8261	48.49	48.49	1.92	0.1891
X_3_-electrolysis time	596.50	596.50	15.54	< 0.05	44.61	44.61	1.77	0.2066
X_4_-settlement time	273.51	273.51	7.12	< 0.05	32.66	32.66	1.29	0.2759
X_1_ X_2_	171.19	171.19	4.46	0.0547	88.45	88.45	3.50	0.0839
X_1_ X_3_	3563.15	3563.15	92.80	< 0.05	345.05	345.05	13.66	< 0.05
X_1_ X_4_	234.80	234.80	6.12	< 0.05	42.84	42.84	1.70	0.2153
X_2_ X_3_	111.68	111.68	2.91	0.1119	46.82	46.82	1.85	0.1964
X_2_ X_4_	11.95	11.95	0.3112	0.5864	73.91	73.91	2.93	0.1109
X_3_ X_4_	107.98	107.98	2.81	0.1174	49.20	49.20	1.95	0.1862
X_1_^2^	174.11	174.11	4.53	0.0529	30.40	30.40	1.20	0.2925
X_2_^2^	9.62	9.62	0.2506	0.6250	1.25	1.25	0.0496	0.8272
X_3_^2^	0.8289	0.8289	0.0216	0.8854	0.6101	0.6101	0.0242	0.8789
X_4_^2^	153.33	153.33	3.99	0.0670	10.33	10.33	0.4090	0.5336
Residual	499.16	38.40			328.26	25.25		
Lack of fit	499.16	49.92			327.91	32.79		
Pure error	0.0000	0.0000			0.3524	0.1175		
Cor total	8540.72				1362.10			

Reflecting on Table 6, the A/V or X_1_ exhibited a significant positive linear effect on turbidity (coefficient = 8.77, p < 0.0001), suggesting that an increase in electrode surface area relative to the volume of greywater enhances the coagulation process, thereby facilitating turbidity removal. This can be primarily attributed to the increase in the available sites for electrochemical reactions, which are crucial for the formation of coagulants in situ^51^. The chemical mechanism involves the oxidation of iron electrodes according to the reaction:

3 $$Fe \to {Fe}^{2+}+2{e}^{-}$$

The Fe^2+^ ions produced can further react with water to form ferrous hydroxide, Fe(OH)_2_​, a flocculant that helps in aggregating and settling the suspended particles:

4 $${Fe}^{2+}+ {2H}_{2}O\to Fe{(OH)}_{2}+2{H}^{+}$$

However, the quadratic term for the A/V ratio (X_1_^2^) was also significant (coefficient = − 8.22, p = 0.053), indicating the presence of an optimum point beyond which additional increases in the A/V ratio may not result in further turbidity reductions. The electrolysis time (X_3_) demonstrated a significant positive impact on turbidity removal (coefficient = 5.76, p = 0.0017), highlighting the importance of adequate reaction time for the formation and aggregation of hydroxide flocs^52,53^. These flocs are effective in adsorbing and trapping colloidal and suspended particles from greywater. The formation of flocs can be enhanced by the continued production of Fe^2+^ ions, which gradually oxidize to form ferric hydroxide, Fe(OH)_3_​, a more effective flocculant^9^:

5 $${4[Fe}^{2+}+2{OH}^{-}]+{O}_{2}+{2H}_{2}O\to 4Fe{(OH)}_{3}$$

Nwabanne and Obi (2017) also reported higher turbidity removal efficiency with increasing electrolysis time mainly due to the increase in dissolved ions in the wastewater leading to an increase in flocs formation^52^. However, it should be noted that higher electrolysis time also increases power consumption. Nonetheless, the interaction term between the A/V ratio and electrolysis time (X_1_.X_3_) was significant and negative (coefficient = − 14.92, p < 0.0001), suggesting a complex interplay where prolonged electrolysis may be less effective at higher A/V ratios. Settling time (X_4_) displayed a less pronounced but still significant positive linear relationship with turbidity (coefficient = 3.90, p = 0.0193), indicating that allowing more time for the precipitates to settle can enhance clarity^54^. However, the quadratic term for settling time (X_4_^2^) suggested a potential parabolic effect (coefficient = − 7.71, p = 0.067), where excessively long settling times might not yield additional clarity benefits and could even be detrimental. In addition, longer settling times may require larger treatment volumes or additional settling tanks, impacting the overall footprint and operational costs of the system. The settling time also depends on how fast the sludge settles. Iron electrodes have been shown to produce a more rapid settling of sludge as compared to aluminum electrodes. In addition, Zodi et al. reported that the settling velocity of sludge is also directly influenced by electrolysis time and increases as the electrolysis time increases^55^. Nevertheless, it is noted that increased electrolysis time results in increased power consumption which can ultimately increase the treatment cost^9,56^.

#### COD response factor

For COD removal, the R^2^ value is 75.90% (Table 5), which is lower than that for turbidity but still denotes a substantial proportion of variance explained by the model. This suggests a considerable degree of fit, indicating that the model captures a significant amount of variability in COD removal, though not as strongly as it does for turbidity. The sum of squares for the COD model (1033.84) indicates the extent of variability captured by the model, which is lower compared to turbidity, reflecting the more complex nature of COD removal. The error sum of squares for COD is 328.26, underscoring the presence of unexplained variability. The mean square for COD is 73.85 for the model and 25.25 for the error. The corresponding F-statistic is 2.92, and the p-value is 0.0305, which, while indicating statistical significance, suggests that the COD model has less predictive strength compared to the turbidity model.

Figures 1, 2, 3, 4b illustrate the 3D surface plots depicting the influence of the interactions between the principal parameters selected in this study on COD removal efficiency. In Fig. 1b, the 3D surface plot elucidates the influence of current density and A/V ratio on COD removal percentage. Similar to turbidity removal, COD removal efficiency increases with higher current densities. Additionally, the A/V ratio plays a significant role, with optimal values enhancing the COD removal process. Figure 2b depicts the effect of electrolysis time and A/V ratio on COD removal. The results indicate that increasing the electrolysis time generally improves COD removal efficiency, with the A/V ratio further enhancing this effect, and the highest removal rates are observed at longer electrolysis times and optimal A/V ratios. Subsequently, Fig. 3b shows the influence of settling time and A/V ratio on COD removal. Longer settling times lead to higher COD removal percentages, underscoring the importance of sufficient sedimentation time in the process. The A/V ratio also contributes positively, with the highest removal efficiencies occurring at optimal values. Lastly, Fig. 4b demonstrates the combined effects of settling time and electrolysis time on COD removal. Both parameters positively influence the removal efficiency, with extended times for both electrolysis and settling resulting in the highest COD removal rates, thereby indicating that an adequate balance of both time factors is crucial for optimal COD removal.

The response function for the COD removal efficiency (%) is shown in Eq. (6) in terms of the coded levels of the factors. Equation (6) reflects a more focused approach, including only those terms that showed statistical significance or marginal significance. The term X_1_ (A/V Ratio) was included due to its marginal significance (F-value = 3.47, p-value = 0.0851). The interaction terms X_1_X_3_ and X_1_X_2_ were also included due to their significant (F-value = 13.66, p-value = 0.0027) and marginally significant (F-value = 3.50, p-value = 0.0839) effects, respectively. This approach acknowledges the complexity of COD removal, which involves a variety of organic compounds, and aims to provide a comprehensive yet statistically sound model.

6 $${\text{COD}} {\text{Removal}} {\text{Efficiency}} = {98}.{49} + {2}.{\text{21X}}_{{1}} - {4}.{\text{64X}}_{{{1}}} {\text{X}}_{{3}} + {2}.{\text{35X}}_{{1}} {\text{X}}_{{2}}$$

The interaction term between the A/V ratio and electrolysis time (X_1_X_3_) showed a notably significant negative coefficient (coefficient =  − 4.64, p = 0.003), indicating a diminishing return or even adverse effects when both the A/V ratio and electrolysis time are increased simultaneously beyond certain limits. The negative impact might be due to over-oxidation leading to inefficient use of energy, excessive generation of metal ions leading to saturation and ineffective flocculation, or the formation of complex organo-metallic compounds that are difficult to degrade. In practice, to optimize COD removal, it's crucial to find the balance point where the rate of reaction and time of exposure minimize energy use while maximizing pollutant removal^57–59^. This is a classic case where operational efficiency needs to be balanced against chemical kinetics, and understanding the interaction helps in setting these operational parameters optimally.

Furthermore, the model indicates that the linear terms for individual operational parameters had less influence on the COD removal efficiency compared to their impact on turbidity removal. This could be indicative of the more complex nature of COD reduction, where the degradation of organic pollutants is influenced by multiple, interrelated factors. Previous studies have reported different outcomes with linear terms proving more significant. Bajpai and Katoch (2021) reported high R^2^ values in a statistically fit and significant model for the treatment of real greywater using iron electrodes^60^. High R^2^ values of other similar studies were also provided with linear terms. Similarly, Wang et al. also examined the influence of electrolysis time as an individual operational parameter on COD reduction in laundry wastewater and reported higher COD removals with extended electrolysis time, in particular when ultrasound was incorporated^61^.

## Conclusion

This study has elucidated the intricacies of laundry greywater treatment using iron electrocoagulation, leveraging RSM-CCD to discern the effects of key operational parameters on turbidity and COD removal efficiencies. The physicochemical analysis of laundry greywater revealed a slightly acidic pH level and high turbidity, indicative of a substantial presence of suspended solids and a significant organic load. The metal concentration analysis pointed out the presence of various elements, including potentially hazardous heavy metals. Compared to other greywater matrices, laundry greywater poses unique challenges for treatment due to its chemical complexity, particularly with the presence of synthetic detergents and dyes. From the response function for turbidity removal, a high R^2^ value of 94.16% demonstrated an excellent fit of the model, with the A/V or X_1_ and electrolysis time or X_3_ having notably positive effects on turbidity reduction. However, the interaction between these factors and their respective quadratic terms indicated the presence of optimal levels for each. Notably, an increase in electrode surface area relative to greywater volume significantly enhanced the coagulation process, a finding that underscores the importance of electrode design in treatment systems. Conversely, the model for COD removal, while still significant, captured a lower proportion of the variance (R^2^ of 75.90%). The interaction between A/V ratio and electrolysis time emerged as particularly influential, suggesting that these parameters' synergistic adjustment could substantially improve organic matter degradation, as reflected in COD levels. This underscores the potential for optimizing electrocoagulation parameters to enhance the treatment of chemically complex greywaters. In conclusion, the study's results highlight the importance of carefully balancing operational parameters in iron electrocoagulation to optimize the treatment of laundry greywater. The demonstrated significance of the A/V ratio and electrolysis time, both individually and interactively, provides a clear direction for the design and operational optimization of electrocoagulation systems. Furthermore, the insights gained into the unique challenges presented by laundry greywater contribute to the broader discourse on sustainable water management and offer a foundation for the development of tailored treatment solutions for greywater reuse applications. Future work should aim to expand upon these findings, exploring the potential of incorporating advanced oxidation processes post-electrocoagulation, examining the effects of electrode materials, and considering the integration of these systems into larger-scale wastewater treatment frameworks. The goal will be to enhance the sustainability and efficacy of greywater recycling systems, contributing to the conservation of water resources and the reduction of environmental impact.

## Data Availability

All data generated or analyzed during this study are included in this published article.
